# Development, validation, and clinical application of an FIA‐MS/MS method for the quantification of lysophosphatidylcholines in dried blood spots

**DOI:** 10.1002/jcla.24099

**Published:** 2021-11-17

**Authors:** Xiaofei Yue, Wei Liu, Ying Liu, Min Shen, Yanhong Zhai, Zhijun Ma, Zheng Cao

**Affiliations:** ^1^ Department of Laboratory Medicine Beijing Obstetrics and Gynecology Hospital Capital Medical University Beijing China; ^2^ Beijing Maternal and Child Health Care Hospital Beijing China; ^3^ Reference Laboratory Medical System Biotechnology Co., Ltd Ningbo Zhejiang China; ^4^ Center of Clinical Mass Spectrometry Beijing Obstetrics and Gynecology Hospital Capital Medical University Beijing China

**Keywords:** flow injection analysis–tandem mass spectrometry, lysophosphatidylcholines, newborn screening, sssdried blood spots

## Abstract

**Background:**

Lysophosphatidylcholine (LPC) plays pivotal roles in several physiological processes and their disturbances are closely associated with various disorders. In this study, we described the development and validation of a reliable and simple flow injection analysis–tandem mass spectrometry (FIA‐MS/MS)‐based method using dried blood spots (DBS) for quantification of four individual LPC (C20:0, C22:0, C24:0, and C26:0).

**Methods:**

Lysophosphatidylcholines were extracted from 3.2 mm DBS with 85% methanol containing 60 ng/ml internal standard using a rapid (30 min) and simple procedure. The analytes and the internal standard were directly measured by triple quadrupole tandem mass spectrometry in multiple reactions monitoring mode via positive electrospray ionization.

**Results:**

Method validation results showed good linearity ranging from 50 to 2000 ng/ml for each LPC. Intra‐ and inter‐day precision and accuracy were within the acceptable limits at four quality control levels. Recovery was from 70.5% to 107.0%, and all analytes in DBS were stable under assay conditions (24 h at room temperature and 72 h in autosampler). The validated method was successfully applied to assessment of C20:0‐C26:0LPCs in 1900 Chinese neonates. C26:0‐LPC levels in this study were consistent with previously published values.

**Conclusion:**

We propose a simple FIA‐MS/MS method for analyzing C20:0‐C26:0LPCs in DBS, which can be used for first‐tier screening.

## Graphical abstract

We proposed a reliable and simple flow injection analysis–tandem mass spectrometry (FIA‐MS/MS)‐based method using dried blood spots (DBS) for quantification of four individual LPC (C20:0, C22:0, C24:0, and C26:0). The elaborated method was successfully applied to assessment of C20:0‐C26:0LPCs in 1900 Chinese neonates.

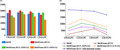

## 1 INTRODUCTION

Lysophosphatidylcholine (LPC), a class of glycerophospholipids, is an important component of human plasma, and is produced by the cleaving of phosphatidylcholine (PC) and/or the transfer of fatty acids to free cholesterol.^1^ As extracellular medium, LPC has a variety of cellular functions including regulating cell proliferation, apoptosis inhibition, and tumor cell invasion, affecting blood vessels, and the nervous systems.^2^, ^3^ Accumulated evidence suggested that abnormal LPC levels were closely correlated with many human diseases, such as diabetes, cancer, cardiovascular, and neurodegenerative diseases.^4^ For example, 26:0‐LPC is known to be elevated in patients and has been applied to screen X‐linked adrenoleukodystrophy (X‐ALD) and other peroxisomal disorders in the neonates.^5^ Consequently, the analysis of LPCs as biomarkers of different disease states has gained considerable interest recently.

Various methods have been developed to detect and measure LPCs in liquid blood.^6^, ^7^, ^8^ Therefore, a few milliliters of blood are generally required to be drawn by venipuncture, while it poses an obvious challenging task to implement in the pediatric populations. In addition to collecting venous blood, dried blood spot (DBS) technique offers a more convenient and less‐invasive option where a small volume of capillary blood (<100 µl) is acquired to a filter paper by a finger prick without massive blood collection.^9^, ^10^ Some of the advantages, such as minimal invasiveness, simplified sample collection, as well as reduced blood volume requirement, facilitate DBS to be most popular among clinics, especially in the laboratory workup for pediatric population.^11^, ^12^ Despite the potential benefits of DBS sampling, highly sensitive analytical methods are required for analysis to overcome the limitation from the small size of the samples. The common technique adopted in DBS is flow injection analysis–tandem mass spectrometry (FIA‐MS/MS). FIA‐MS/MS is a simple and fast method, in which samples are introduced to the MS instrument directly without including any chromatographic system.^13^, ^14^ This allows the rapid measurement of multiple analytes with high sensitivity and specificity. In view of this, FIA‐MS/MS combined with DBS sampling has been rated as one of the most cost‐ and time‐effective strategies and widely utilized for high‐throughput screening.

However, the reports of FIA‐MS/MS methods for the quantification of LPCs in DBS samples are limited. Turgeon et al. initially demonstrated the use of DBS technology combined with FIA‐MS/MS method for estimating LPCs in 2015.^15^ A panel of LPCs (C20:0, C22:0, C24:0, and C26:0) was also analyzed in India by Natarajan et al. with a reliable and fast FIA‐MS/MS method, where they measured the concentrations and the ratios of LPC.^5^ Except for Tian’s work adopting the NeoBase^TM^ 2 Non‐derivatized MSMS kit, no further reported was published on DBS‐based assay for measuring LPCs with Chinese neonates.^16^ NeoBase^TM^ 2 kit was an upgraded version of NeoBase^TM^ 1 and it added more metabolites including 3 amino acids, 5 acylcarnitines, 4 lysophospholipids, 2 nucleosides, and the coverage was expanded to 57 metabolites. Although the use of commercial kit might be preferable for simultaneous quantification of various metabolites in clinical laboratories, it would be costly when measuring a portion of targeted compounds.

The aim of this study was to develop a first‐tier method for simultaneous and high‐throughput quantification of four LPCs (C20:0, C22:0, C24:0, and C26:0) by combining DBS sampling with FIA‐MS/MS. After the bioanalytical validation, the method was applied to a collection of clinical samples of 1900 Chinese newborns to further explore its clinical utility.

## 2 MATERIALS AND METHODS

### 2.1 Chemicals and reagents

The chemical standards of C20:0, C22:0, C24:0, and C26:0‐LPC and isotope labeled internal standard C26:0‐d4‐LPC were purchased from Avanti Polar Lipids, Inc. HPLC‐MS grade methanol and acetonitrile were purchased from Merck. Analytical grade chloroform, formic acid, and ammonium acetate were purchased from Sigma‐Aldrich. Filter paper grade 903 was obtained from Whatman GmbH.

### 2.2 DBS preparation

Independent primary stock solutions of four LPCs were prepared at a concentration of 1 mg/ml in methanol. Primary stock solutions in methanol were then mixed to prepare pooled standard stock solution and stored at −20℃. Working solutions for calibration standards were obtained by diluting the standard stock solution, resulting in a series of solutions with concentrations of 2500, 6250, 12,500, 25,000, 50,000, and 100,000 ng/ml for LPCs. DBS for calibration standards and QC samples were prepared as described below. Calibration standards were obtained by diluting the working standard 50‐fold with pooled EDTA blood to the concentrations between 50 to 2000 ng/ml. The QC samples at 50, 125, 500, and 1000 ng/ml were also prepared similarly. An aliquot (50 µl) of spiked blood was subsequently spotted on filter paper card and dried overnight at room temperature. All the DBS cards were finally stored at −20℃ in ziplock bags with desiccant.

### 2.3 Sample extraction

For each sample, a single 3.2 mm disk was punched from the DBS card into a 96‐well microtiter plate and 100 µl of extraction working solution (85% aqueous methanol) containing deuterated internal standard (60 ng/ml C26:0‐d4‐LPC) was added. Then, the plate was covered and shaken at 450 rpm for duration of 30 min at 45℃. The extract from each well was then transferred to a fresh 96‐well plate and sealed with heat sealing foil for FIA‐MS/MS analysis.

### 2.4 FIA‐MS/MS analysis

Quantitative analysis was conducted using Waters Xevo TQD triple quadrupole mass spectrometer (Water Corporation) with an electrospray ionization (ESI) source operated in positive ion mode. Analytes and the internal standard were monitored in the multiple reactions monitoring (MRM) mode with the following quantification ion pairs 636.5 > 104.0 (C26:0‐LPC), 608.5 > 104.1 (C24:0‐LPC), 580.4 > 104.1 (C22:0‐LPC), 552.4 > 104.1 (C20:0‐LPC), and 640.6 > 104.1 (C26:0‐d4‐LPC). The optimized ionization source parameters were as follows: capillary voltage 3.0 kV, cone voltage 55 V, source temperature 150℃, desolvation temperature 350℃.

The injection volume was 10 µl for each sample. The mobile phase comprising methanol/water (85/15, v/v) with 5 mM ammonium acetate was used for elution. The initial flow rate was 0.2 ml/min, and then reduced to 0.02 ml/min between 0.12 and 1.0 min, increased to 0.8 ml/min between 1.0 and 1.2 min, and finally returned to 0.2 ml/min between 1.2 and 2.0 min. The concentration of LPCs (C20:0, C22:0, C24:0, and C26:0) was calculated using the following formula: (analyte peak area)/(internal standard peak area) × (concentration of internal standard) × dilution factor. Dilution factor was 31.25, which represents the dilution of blood from the 3.2 mm DBS disk in 100 µl of extraction solution.

### 2.5 Method validation

Linearity of the assays was assessed from a calibration curve established by plotting the peak–area ratios of analyte to IS vs. the concentration ranging from 50 to 2000 ng/ml. The calibration function was fitted by linear regression model and the coefficient of determination (*R* ^2^) was calculated.

Accuracy and precision were evaluated by repeatedly analyzing QC samples at four concentration levels with 10 replicates on the same day (intra‐day) and between three consecutive days (inter‐day). Accuracy was calculated from the percent difference between the measured concentration and the nominal concentration. Precision was expressed as the percentage coefficient of variation (%CV).

Recovery was evaluated by analyzing DBS samples before and after the addition of QC standards and determined by measuring the ratio of the increased concentration to the added concentration at four different concentrations.

Stability experiments were performed by repeated analysis QC samples and presented as percent of the concentration originally measured, and the storage conditions were as follows: (1) room temperature for 24 h prior to pretreatment as bench‐top stability, (2) autosampler (4 °C) for 72 h as postpreparative stability.

To investigate the effects of the hematocrit (Hct) of whole blood on quantification, fresh blood was centrifuged and the red blood cells were washed three times with saline. Then, different volumes of plasma and red blood cells were mixed to obtain blood samples with different Hct levels (0.30, 0.40, 0.5, and 0.6). QC concentrations at 50, 125, 500, and 1000 ng/ml were tested at four Hct Levels.

### 2.6 Application to clinical samples

This study was reviewed and approved by Beijing Obstetrics and Gynecology Hospital Research Ethics Committee. A total of 1900 anonymous samples were obtained from residual newborn DBS samples following routine newborn screening between April 2020 and June 2020 in Beijing Newborn Screening Center. All participants were negative for screening programs. DBS were collected by heel prick method between 24 h and 7 days after birth and spotted on Whatman filter paper cards for analysis.

### 2.7 Statistical analysis

Quantification was performed using Masslynx 4.2 employing the Neolynx^TM^ program. Statistical analysis was performed using SPSS 26.0 software (SPSS Inc.). Reference intervals for each analyte were expressed as medians (50th) and the 1st and 99.9th percentiles of the distributions.

## 3 RESULTS AND DISCUSSION

### 3.1 Method optimization

#### 3.1.1 Extraction procedure

**FIGURE 1:**
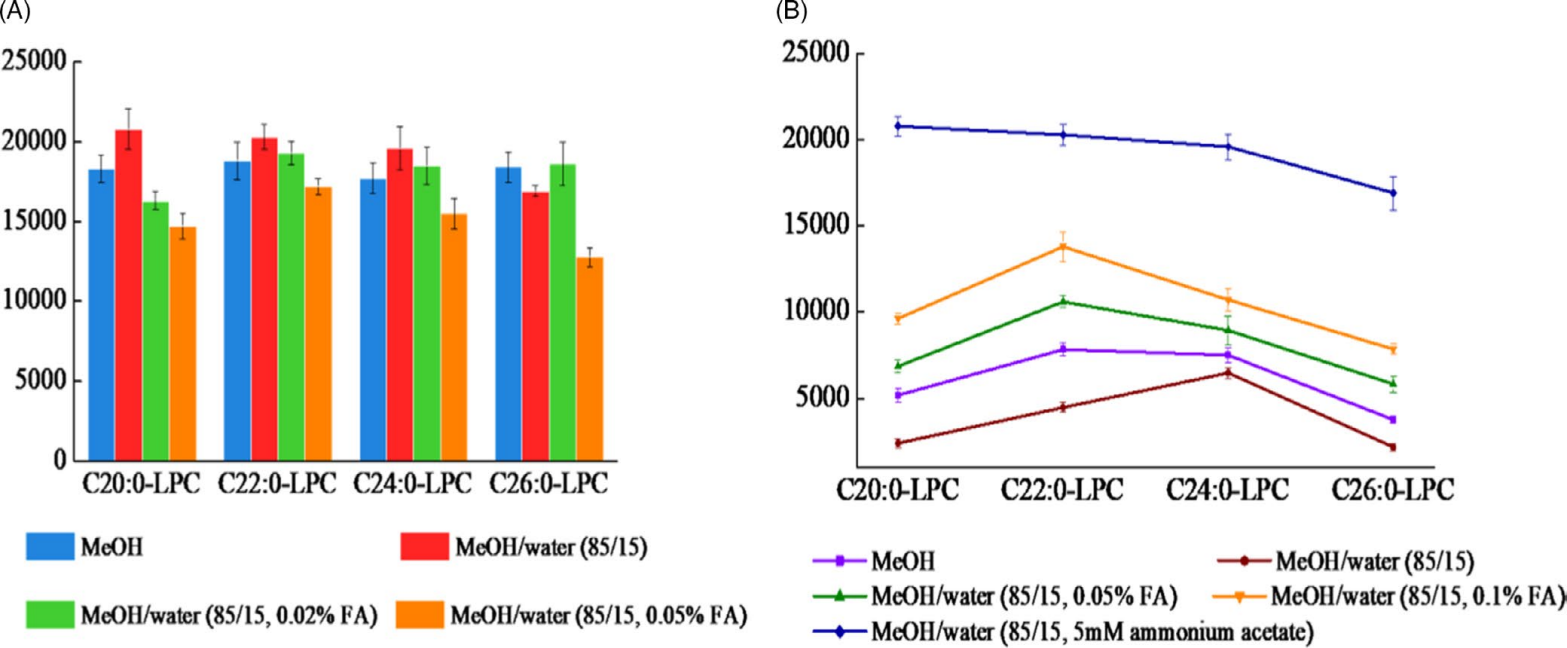
(A) Optimization of the extraction solvent; and (B) mobile phase for the analysis of the LPCs

A simple and efficient extraction procedure is important for first‐tier method in order to minimize sample turn‐around time and maintain extraction sufficiency. In general, extraction methods for the analysis of DBS samples consist of several steps: punching out a disk, adding extraction solvent (such as methanol, acetonitrile, or mixture) and derivatization (if necessary), then shaking, vortexing, or sonification.^17^ Similar sample preparation strategy was also adopted in our method. Five extraction solvents including methanol, a mixture of methanol/water (85/15, v/v), a mixture of 0.02% formic acid in methanol/ water (85/15, v/v), and a mixture of 0.05% formic acid in methanol/water (85/15, v/v), were evaluated. The extraction profile of four analytes at various conditions is shown in Figure 1. Basically, the signal intensity of LPCs was considerably increased in the presence of water for most of the analytes. The addition of formic acid had little positive effect on single response, and too much formic acid was found to yield decreased single on the contrary. A mixture of methanol/water (85/15, v/v) produced the best extract effect with highest signal response.

In addition, a shaking incubation step was included because of its reduced extraction time and improved extraction efficiency. Experiments were also conducted to determine optimal incubation conditions by testing different incubation time (30, 45, and 60 min), incubation temperature (room temperature, 45℃), and shaking speed (450, 750 rpm). No significant difference was observed with respect to the extraction efficiency. Nevertheless, 45℃ was selected as the optimal temperature because it is easy to control. Therefore, the optimal values regarding incubation procedure were 30 min, 45℃, and 450 rpm.

#### 3.1.2 FIA‐MS/MS conditions

Subsequently, the elution conditions were further investigated. For the mobile phase composition, methanol, the mixture of methanol and water was tested, with formic acid (0.05%, 0.1%), ammonium acetate (5 mM) used as an additive under all conditions. It was found that addition of ammonium acetate could provide higher peak intensity than formic acid (Figure 1). However, when the ammonium acetate and formic acid added together, no extra signal enhancement was observed. Therefore, methanol/water (85/15, v/v) with 5 mM ammonium acetate was selected as the mobile phase.

In terms of the elution gradient, both constant flow rate and variable flow rate elution were evaluated. In the initial stage of method development, analytes in neat solutions were able to acquire symmetrical peaks using constant flow rate; however, chromatographic peak shapes from DBS extraction became bifurcation and tailing. It may be due to the reason that the entire sample injection band is introduced at the same time to the mass spectrometer in the FIA‐MS/MS run; thus, analytes of interest are not separated from endogenous compounds which could lead to interferences.^13^, ^18^ Consequently, we switched to variable flow rate that started with a rapid flow rate followed by a reduced rate to obtain a wide chromatographic peak and form a relative steady state for analysis.

After the evaluation of a series of parameters that influence extraction process and FIA‐MS/MS method, we finally exploited a robust and fast FIA‐MS/MS quantification method for LPCs by skipping chromatographic separation. The major advantages of the present method are that the analysis requires only 3.2 mm DBS cards and it has a short run‐time (2 min), facilitating it as a potential first‐tier method for high‐throughput screening. Sample treatment is relatively quick and simple, and only involves methanol extraction, ensuring the feasibility in clinical practice.

### 3.2 Method performance

Linearity of the method was assessed using 6‐point calibration curve selected to cover the range of expected physiological concentrations of each analyte. With the DBS calibrators, the method showed good linearity in the range of 50–2000 ng/ml for all the analytes. The correlation coefficient (*R* ^2^) determined by least‐squares analysis was as follows: 0.998 for C20:0‐LPC, 0.997 for C22:0‐LPC, 0.998 for C24:0‐LPC, and 0.999 for C26:0‐LPC.

The methods were evaluated in terms of intra‐day accuracy and precision by assaying QC samples. As shown in Table 1, the intra‐day precision for all compounds was less than 14% and accuracy ranges from 85.0% to 115.8% (Table 1). The inter‐day precision was assessed by analyzing three sets of all QC samples on three consecutive days. The inter‐day CV was less than 16% with the corresponding accuracy of 85.4%–115% (Table 1). This method displayed satisfactory precision and accuracy meeting acceptance criteria for bioanalytical methods.

**TABLE 1:** Precisions and accuracies of the method (*n *= 10)

LPCs	Spiked concentration (ng/ml)	Inter‐day concentration (ng/ml)	CV (%)	Accuracy (%)	Intra‐day concentration (ng/ml)	CV (%)
C26:0‐LPC	50	48	17.58	96.32	50	16.78
	125	116	7.08	93.07	116	10.51
	500	483	7.05	96.59	490	7.69
	1000	1025	3.95	102.53	1066	6.70
C24:0‐LPC	50	51	18.24	101.56	50	17.92
	125	120	7.21	96.07	117	9.72
	500	524	5.s66	104.84	512	7.09
	1000	1118	4.50	111.80	1136	5.42
C22:0‐LPC	50	55	16.13	110.18	53	15.63
	125	119	5.65	95.00	115	9.22
	500	522	6.03	104.49	503	7.52
	1000	1060	4.76	105.97	1063	5.49
C20:0‐LPC	50	51	13.86	101.32	50	14.86
	125	115	6.41	91.95	109	10.95
	500	487	4.28	97.36	463	6.96
	1000	989	5.06	98.90	988	6.13

Recovery was determined by analyzing DBS samples prepared from pooled blood with known analyte concentrations before and after the spiking of the four levels of QC standards. In this study, the recovery was 88.4%–106.83% at the concentrations of 125, 500, and 1000 ng/ml (Table 2), but lower than 80% at the concentration of 50 ng/ml owing to the endogenous levels are higher than the spiked concentration.

**TABLE 2:** Recovery of LPCs (*n* = 6)

LPCs	Endogenous concentration (ng/ml)	Spiked concentration (ng/ml)	Measured concentration (ng/ml)	Recovery (%)
C26:0‐LPC	129	50	165	73.22
		125	252	99.04
		500	630	100.04
		1000	1111	98.20
C24:0‐LPC	136	50	173	74.58
		125	263	102.33
		500	658	104.57
		1000	1204	106.83
C22:0‐LPC	146	50	139	70.52
		125	221	93.29
		500	623	103.70
		1000	1112	101.10
C20:0‐LPC	77	50	115	75.83
		125	187	88.42
		500	567	98.07
		1000	1021	94.45

The stability of LPCs before and after pretreatment samples was tested under a variety of conditions using QC samples. The stability results (Table S1) showed that no significant degradation of analytes in DBS samples was observed when stored at room temperature for 24 h. Additionally, the stability of processed sample stability in the autosampler over a 72 h period was also evaluated. The results suggested that all processed samples were stable in the autosampler for 72 h. The average accuracy of each concentration was within ±15% of its originally measured value.

Blood samples with different Hct values might affect the spotting area, spot composition, and quantification results. Hct values usually range between 49% and 61% for newborns. To investigate the effects of Hct, four Hct levels (0.3, 0.4, 0.5, and 0.6) and at QC concentrations of 50, 125, 500, and 1000 ng/ml were analyzed. The accuracies at higher Hct levels (0.4, 0.5, and 0.6) were 81.2–115.2%; however, the accuracy was lower than 80% for C26:0‐LPC at the lower Hct level (0.3) (Table S2). In summary, limited effect of hematocrit on LPCs concentration in DBS samples was observed.

### 3.3 Clinical application

Next, the developed method was applied to 1900 DBS samples from the newborns at our institute for the measurement of LPC concentrations and their relative rations. Their mean values with standard deviation and the median along with concentration range were shown in Table 3. The earlier publication of C20:0‐C26:0‐LPCs levels in American newborns reported normal median values of 140, 90, 190, and 180 ng/ml with a small sample size.^15^ A similar‐scaled study was conducted in India population aged 1–60 years and the four LPCs levels were 0.32, 0.17, 0.29, and 0.16 µM.^5^ Tian et al conducted a new born screening study for determining C20:0‐C26:0‐LPCs levels in Shanghai population, involving 3078 DBS specimens. The median values of LPCs were 0.18, 0.14, 0.37, and 0.20 µM, respectively.^17^ With respect to the median value of C26:0‐LPC and C24:0‐LPC, our results were in good agreement with those reported by Tian et al, while not consistent with the other two studies especially for C22:0‐LPC and C20:0‐LPC. This discrepancy might be due to difference of sample size, age, ethnicity, and region.

**TABLE 3:** Concentration ranges of LPCs in newborns

Analytes	Mean concentration (standard deviation) in newborns (ng/ml)	Median and concentration range in newborns (ng/ml)^a^
C26:0‐LPC	132 (48)	120 (68–292)
C24:0‐LPC	230 (53)	223 (125–373)
C22:0‐LPC	159(43)	153 (83–288)
C20:0‐LPC	165 (62)	153 (74–398)

Analyte ratios	Mean (standard deviation) in newborns	Median and range in newborns
C26:0/C22:0‐LPC	0.85 (0.24)	0.81 (0.43–1.71)
C26:0/C20:0‐LPC	0.87 (0.35)	0.82 (0.33–2.00)
C24:0/C22:0‐LPC	1.47 (0.19)	1.47 (1.02–1.95)
C24:0/C20:0‐LPC	1.49 (0.40)	1.46 (0.76–2.63)

^a^ Median (range is expressed between 1st and 99th percentile).

X‐linked adrenoleukodystrophy is the most common inherited peroxisomal disorder caused by mutations in the ABCD1 gene. The disease is characterized by an obstacle of beta‐oxidation, resulting in very long‐chain fatty acid accumulation in plasma and tissues.^19^ Recent studies have demonstrated that elevated C26:0‐LPC is a sensitive and specific biomarker which could be used for screening of X‐ALD and other peroxisomal disorders. With the advancements in early screening, diagnosis, and treatment, X‐ALD was added to the federal recommended uniform screen panel in 2016. New York became the first state in the United States to begin universal newborn screening for X‐ALD screening by examining C26:0‐LPC values, followed by Connecticut and California.^20^, ^21^ Although screening for X‐ALD has not yet been implemented in China, there are increasing provinces and regions that are willing to expand X‐ALD as a routine newborn screening program in the near future. The presented approach displayed reliable quantitation of four different LPCs in DBS with minimum pretreatment.

## 4 CONCLUSIONS

In this study, we described a fast, reliable, and simple method for determination of LPCs in DBS by FIA‐MS/MS with ESI positive ionization. The method allowed the analysis of maximum thirty samples per hour. The straightforward samples preparation procedure can be applied in a high throughput manner. In addition, DBS sampling provided more convenient and comfortable way for special populations such as critically ill and infants. In this way, it was beneficial to analyze the vast majority of samples in the clinical practice with this method, which was time‐ and cost‐effective. The developed method was fully validated in terms of the linear range, accuracy, precision, recovery, stability, and Hct and has been successfully applied to the real sample analysis. In view of the pivotal roles of LPCs in various diseases, we believe that the utility of the method is not limited to X‐ALD newborn screening application but also has potential to be extended to predict the severity of many others. However, more rigorous clinical evaluation of the present method needs to be carried out in the future to further verify its utility in various clinical scenarios.

## DATA AVAILABILITY STATEMENT

The data during and/or analyzed during the current study are available from the corresponding author on reasonable request.

## Supporting information

Table S1‐S2

## References

[1] Law SH , Chan ML , Marathe GK , et al. An updated review of lysophosphatidylcholine metabolism in human diseases. Int J Mol Sci. 2019;20:1149. 10.3390/ijms20051149 PMC6429061 30845751

[2] Liu JN , Li J , Li SN , et al. Circulating lysophosphatidylcholines in early pregnancy and risk of gestational diabetes in Chinese women. J Clin Endocrinol Metab. 2020;105:e982‐e993. 10.1210/clinem/dgaa058 32016391

[3] Liu H , Liu N , Teng WF , Chen J . Study on a dSPE‐LC‐MS/MS method for lysophosphatidylcholines and underivatized neurotransmitters in rat brain tissues. J Chromatogr B. 2018;1096:11‐19. 10.1016/j.jchromb.2018.07.040 30125781

[4] Liu PP , Zhu W , Chen C , et al. The mechanisms of lysophosphatidylcholine in the development of diseases. Life Sci. 2020;247:117443. 32084434 10.1016/j.lfs.2020.117443

[5] Natarajan A , Christopher R , Netravathi M , Bhat M , Chandra SR . Flow injection ionization‐tandem mass spectrometry‐based estimation of a panel of lysophosphatidylcholines in dried blood spots for screening of X‐linked adrenoleukodystrophy. Clin Chim Acta. 2019;495:167‐173. 30980791 10.1016/j.cca.2019.04.059

[6] Takatera A , Takeuchi A , Saiki K , Morisawa T , Yokoyama N , Matsuo M . Quantification of lysophosphatidylcholines and phosphatidylcholines using liquid chromatography‐tandem mass spectrometry in neonatal serum. J Chromatogr B. 2006;838:31‐36. 10.1016/j.jchromb.2006.03.006 16603422

[7] Gardner MS , Kuklenyik Z , Lehtikoski A , et al. Development and application of a high throughput one‐pot extraction protocol for quantitative LC‐MS/MS analysis of phospholipids in serum and lipoprotein fractions in normolipidemic and dyslipidemic subjects. J Chromatogr B. 2019;1118–1119:137‐147. 10.1016/j.jchromb.2019.04.041 PMC7138451 31035135

[8] Peng ZX , Zhang Q , Mao ZM , et al. A rapid quantitative analysis of bile acids, lysophosphatidylcholines and polyunsaturated fatty acids in biofluids based on ultraperformance liquid chromatography coupled with triple quadrupole tandem massspectrometry. J Chromatogr B. 2017;1068–1069:343‐351. 10.1016/j.jchromb.2017.10.066 29129603

[9] Burnett J . Dried blood spot sampling: practical considerations and recommendation for use with preclinical studies. Bioanalysis. 2011;3:1099‐1107. 21585305 10.4155/bio.11.68

[10] Tey HY , See HH . A review of recent advances in microsampling techniques of biological fluids for therapeutic drug monitoring. J Chromatogr A. 2021;1635:461731. 33285415 10.1016/j.chroma.2020.461731

[11] Enderle Y , Foerster K , Burhenne J . Clinical feasibility of dried blood spots: analytics, validation, and applications. J Pharm Biomed Anal. 2016;130:231‐243. 27390013 10.1016/j.jpba.2016.06.026

[12] Avataneo V , D’Avolio A , Cusato J , Cantù M , De Nicolò A . LC‐MS application for therapeutic drug monitoring in alternative matrices. J Pharm Biomed Anal. 2019;166:40‐51. 30609393 10.1016/j.jpba.2018.12.040

[13] Nanita SC , Kaldon LG . Emerging flow injection mass spectrometry methods for high‐throughput quantitative analysis. Anal Bioanal Chem. 2016;408:23‐33. 26670771 10.1007/s00216-015-9193-1

[14] Taki K , Noda S , Hayashi Y , Tsuchihashi H , Ishii A , Zaitsu K . A preliminary study of rapid‐fire high‐throughput metabolite analysis using nano‐flow injection/Q‐TOFMS. Anal Bioanal Chem. 2020;412:4127‐4134. 32328692 10.1007/s00216-020-02645-1

[15] Turgeon CT , Moser AB , Mørkrid L , et al. Streamlined determination of lysophosphatidylcholines in dried blood spots for newborn screening of X‐linked adrenoleukodystrophy. Mol Genet Metab. 2015;114:46‐50. 25481105 10.1016/j.ymgme.2014.11.013

[16] Tian GL , Xu F , Jiang K , Wang YM , Ji W , Zhuang YP . Evaluation of a panel of very long‐chain lysophosphatidylcholines and acylcarnitines for screening of X‐linked adrenoleukodystrophy in China. Clin Chim Acta. 2020;503:157‐162. 31978407 10.1016/j.cca.2020.01.016

[17] Andrlova L , Kandar R . The dried blood spot sampling method in the laboratory medicine. Bratisl Med J. 2019;120:223‐234. 10.4149/BLL_2019_035 31023042

[18] Ciasca B , Pecorelli I , Lepore L , et al. Rapid and reliable detection of glyphosate in pome fruits, berries, pulses and cereals by flow injection – Mass spectrometry. Food Chem. 2020;310:125813. https://doi:1016/j.foodchem.2019.125813 31757486 10.1016/j.foodchem.2019.125813

[19] Engelen M , Kemp S , Poll‐The BT . X‐Linked adrenoleukodystrophy: pathogenesis and treatment. Curr Neurol Neurosci Rep. 2014;14:486. 25115486 10.1007/s11910-014-0486-0

[20] Moser AB , Jones RO , Hubbard WC , et al. Newborn screening for X‐linked adrenoleukodystrophy. Int J Neonatal Screen. 2016;2:15. 31467997 10.3390/ijns2040015 PMC6715319

[21] Vogel BH , Bradley SE , Adams DJ , et al. Newborn screening for X‐linked adrenoleukodystrophy in New York State: diagnostic protocol, surveillance protocol and treatment guidelines. Mol Genet Metab. 2015;114:599‐603. 25724074 10.1016/j.ymgme.2015.02.002

