# Supplementary material for: Development, validation, and clinical application of an FIA‐MS/MS method for the quantification of lysophosphatidylcholines in dried blood spots
Source: J Clin Lab Anal. 2021 Nov 17;36(1):e24099. doi: 10.1002/jcla.24099 (PMC8761423; doi:10.1002/jcla.24099)
Supplement: Supplementary file 1 — Table S1‐S2 [file JCLA-36-e24099-s001.docx]

**Supporting information**

**Development, validation and clinical application of an FIA-MS/MS method for the quantification of lysophosphatidylcholines in dried blood spots**

Xiaofei Yue^1, 2^, Wei Liu^1, 2^, Ying Liu^1, 2^, Min Shen^3^, Yanhong Zhai^1, 2^, Zhijun Ma^1, 2*^, Zheng Cao^1, 2, 4*^

^1^ Department of Laboratory Medicine, Beijing Obstetrics and Gynecology Hospital, Capital Medical University, Beijing, China

^2^ Beijing Maternal and Child Health Care Hospital, Beijing, China

^3^ Reference Laboratory, Medical System Biotechnology Co., Ltd, Ningbo, Zhejiang, China

^4^ Center of Clinical Mass Spectrometry, Beijing Obstetrics and Gynecology Hospital, Capital Medical University, Beijing, China

^*^ Co-corresponding authors:

Zheng Cao, [zhengcao2011@ccmu.edu.cn](mailto:zhengcao2011@ccmu.edu.cnT), Tel: +86-10-52276406

Zhijun Ma, [masu7162000@hotmail.com](mailto:masu7162000@hotmail.com), Tel: +86-10-52276506

Address: Department of Laboratory Medicine, Beijing Obstetrics and Gynecology Hospital,

**Table S-1 Stability of LPCs at room temperature and at autosampler**

**Table S-2 Impact of hematocrit on accuracy in DBS**

**TABLES**

**Table S-1** Stability of LPCs at room temperature and at autosampler

| **LPCs** | **Concentration** | **Room temperature for 24h** |  | **4℃ for 72h** |  |
| --- | --- | --- | --- | --- | --- |
|  | **(ng/mL)** | **Accuracy (%)** | **CV (%)** | **Accuracy (%)** | **CV (%)** |
| **C26:0-LPC** | 50 | 112.43 | 5.82 | 98.78 | 12.50 |
|  | 125 | 104.77 | 7.65 | 101.59 | 12.61 |
|  | 500 | 100.59 | 10.17 | 100.86 | 7.44 |
|  | 1000 | 99.27 | 4.64 | 100.60 | 4.67 |
| **C24:0-LPC** | 50 | 94.86 | 14.44 | 112.72 | 13.34 |
|  | 125 | 93.41 | 12.26 | 108.83 | 13.78 |
|  | 500 | 89.85 | 2.42 | 110.86 | 2.77 |
|  | 1000 | 97.82 | 2.20 | 109.22 | 2.67 |
| **C22:0-LPC** | 50 | 98.01 | 8.43 | 109.32 | 14.75 |
|  | 125 | 96.73 | 12.05 | 99.18 | 4.39 |
|  | 500 | 90.70 | 2.23 | 107.25 | 3.08 |
|  | 1000 | 97.98 | 3.33 | 102.41 | 4.60 |
| **C20:0-LPC** | 50 | 98.20 | 11.08 | 106.11 | 12.47 |
|  | 125 | 99.10 | 11.81 | 96.33 | 7.74 |
|  | 500 | 89.17 | 7.36 | 102.99 | 4.91 |
|  | 1000 | 92.60 | 2.29 | 106.02 | 2.64 |

**Table S-2** Impact of hematocrit on accuracy in DBS

| **LPCs** | **Norminal concentration** | **HCT（0.3）** | **HCT (0.4)** | **HCT (0.5)** | **HCT (0.6)** |
| --- | --- | --- | --- | --- | --- |
|  | **(ng/mL)** | **Accuacy (%)** | | | |
| **C26:0-LPC** | 50 | 79.8 | 100.0 | 99.0 | 105.1 |
|  | 125 | 92.9 | 90.5 | 110.8 | 101.9 |
|  | 500 | 102.1 | 107.1 | 114.6 | 109.4 |
|  | 1000 | 94.1 | 102.1 | 104.7 | 105.6 |
| **C24:0-LPC** | 50 | 96.8 | 117.0 | 100.8 | 110.0 |
|  | 125 | 92.8 | 97.3 | 106.4 | 113.1 |
|  | 500 | 102.2 | 110.7 | 111.6 | 114.7 |
|  | 1000 | 98.9 | 111.8 | 109.7 | 115.8 |
| **C22:0-LPC** | 50 | 88.6 | 113.9 | 99.1 | 104.4 |
|  | 125 | 86.2 | 94.5 | 101.5 | 98.5 |
|  | 500 | 91.3 | 101.7 | 99.5 | 107.0 |
|  | 1000 | 99.6 | 105.0 | 102.0 | 109.6 |
| **C20:0-LPC** | 50 | 90.3 | 113.0 | 108.4 | 106.8 |
|  | 125 | 95.8 | 93.1 | 103.4 | 106.4 |
|  | 500 | 91.5 | 99.2 | 100.0 | 104.5 |
|  | 1000 | 97.1 | 104.9 | 101.9 | 109.2 |
